# Genetic associations of corneal astigmatism in Hong Kong Chinese children

**DOI:** 10.1038/s41598-026-46723-3

**Published:** 2026-03-31

**Authors:** Erica Shing, Ka Wai Kam, Yu Yao Wang, Yuzhou Zhang, Xiu Juan Zhang, Pancy O. S. Tam, Alvin L. Young, Clement C. Tham, Chi Pui Pang, Jason C. Yam, Li Jia Chen

**Affiliations:** 1 Department of Ophthalmology and Visual Sciences, The Chinese University of Hong Kong, Hong Kong, China; 2 Department of Ophthalmology and Visual Sciences, Prince of Wales Hospital, Hong Kong, China; 3 Hong Kong Eye Hospital, Hong Kong, China; 4 Hong Kong Hub of Paediatric Excellence, The Chinese University of Hong Kong, Hong Kong, China; 5 Department of Ophthalmology, The University of Hong Kong, Hong Kong, China; 6 Department of Ophthalmology and Visual Sciences, The Chinese University of Hong Kong, Hong Kong Eye Hospital, 147K, Argyle Street, Kowloon, Hong Kong, China

**Keywords:** Genetics, Single-nucleotide polymorphism, Astigmatism, Refractive error, Children, Diseases, Genetics, Medical research, Risk factors

## Abstract

This study aims to investigate the associations of multiple single-nucleotide polymorphisms (SNPs) with the risk and magnitude of corneal astigmatism (CA) in children. A total of 14 SNPs were genotyped in 2167 Chinese children aged 4–11 years, recruited from the Hong Kong Children Eye Study. The main outcomes were allelic associations with the risk of significant CA (≥ 1.0D) and magnitude of CA. Allelic associations with the risk of significant CA and magnitude of CA, respectively, were evaluated in additive model through age- and sex-adjusted logistic and linear regression. Subgroup analysis included sex-stratified analysis and linear trend test across age. *FMNL2* rs1579050 was associated with the magnitude of CA (β = 0.158D, Pc = 0.045), and with the magnitude of CA (β = 0.301D, Pc = 0.02) in age quartile 4 in the age subgroup analysis. *PDGFRA* rs17084051 (*P* = 0.04) and *ZC3H11B* rs7525202 (*P* = 0.01) were associated with linear decrease in CA magnitude across age (*P* < 0.05 considered statistically significant). This study revealed *FMNL2*, *PDGFRA* and *ZC3H11B* as potential genetic factors for corneal astigmatism among Chinese children, with age-specific effects. Further validation of the findings in large cohorts should be warranted.

**Supplementary Information:**

The online version contains supplementary material available at 10.1038/s41598-026-46723-3.

## Introduction

Astigmatism is a prevalent refractive error, affecting 14.9% of children and 40.4% of adults worldwide^1^. The total astigmatism is often referred to as refractive astigmatism (RA), which includes corneal astigmatism (CA) and internal astigmatism (IA). Generally, CA, numerically defined as the difference between the steepest and flattest corneal curvatures (CC), is the major component that determines the type and magnitude of RA, and it can be fully or partially compensated by IA^2^. If left untreated, astigmatism can adversely impact visual acuity^3^ and contrast sensitivity^4^, leading to higher amblyogenic risk^5^ and poorer school performance^6^ in children. Notably, CA is often present in newborns in substantial amounts, particularly in those with low birthweight or low postconceptional age^7^. As children grow, CA generally decreases due to changes in corneal curvature during the process of emmetropization, leading to a reduction in RA^8^ that remains relatively stable until older adulthood. Since CA is the major component of astigmatism, a higher magnitude of CA at a given age may predispose to higher RA, as IA is relatively constant and may not be able to fully compensate for large corneal toricity^8^. Currently, the exact cause underlying CA remains unclear. It is a complex trait influenced by a combination of environmental and genetic factors, resulting in variability among individuals.

Historically, there has been controversy regarding the genetic contribution to CA. Heritability studies showed a higher correlation of CA in monozygotic twins compared to dizygotic twins and among families^9–12^, while some studies reported no differences^13,14^. In recent years, genome-wide association studies (GWAS) for the genetic factors influencing CA found a shared loci with RA and CC^15–17^, and identified genes such as *CASC15*^15,18^, *FMNL2*^15^, and *PDGFRA*^19,20^. *PDGFRA* is known for its association with CC among Asians^21,22^, as well as with CA in Japanese^23^. In a recent meta-analysis of all published GWAS and genetic association studies up to 2023, we identified moderate heritability for RA, CA and CC. We also systematically reviewed 50 single-nucleotide polymorphisms (SNPs) in 10 genes that carry overlapping associations between RA, CA and CC, and found that *CASC15* was associated with all three traits whereas *FMNL2* and *PDGFRA* showed overlapping associations between RA and CA, and between CA and CC, respectively^24^.

Despite the findings from GWAS, replication studies on the genetic associations of CA remained limited, with most primarily concentrating on adult populations. While CA manifests at birth, it is also influenced by environmental factors. Consequently, early variations in CA of a child—which is presumably determined by genetics—may not persist into adulthood due to the cumulative impact of environmental and lifestyle influences. Thus, studying the genetic factors of CA in children may help unravel the genetic force of astigmatism formation early in life, and this study aims to investigate the genetic associations of CA in young children.

## Results

### Demographics of study subjects

This study involved a total of 2,167 children aged 4–11 years (mean age ± SD, 7.67 ± 1.05 years; 54.3% male). Their demographics were summarized in Table S1. Out of the 2,167 children, 1,282 (59.2%) had CA ≥ 1.0D.

## Associations of SNPs with the risk of significant CA and magnitude of CA

All 14 candidate SNPs were successfully genotyped with a call rate of 100%. The genotyping results were summarized in Table S2. No SNPs were found to be associated with the risk of significant CA (Table 1). *FMNL2* rs1579050 was associated with magnitude of CA (G vs. A, β = 0.158D, 95% CI = 0.055–0.262, *P* = 0.0028, *Pc* = 0.04; Table 2).

**Table 1:** Allelic associations of SNPs with the risk of significant Corneal Astigmatism (≥ 1.0D) in 2,167 children.

Chr	Gene/Locus	SNP	A1	OR	95% CI	*P* *	Pc ^#^
4	*BMP3*	rs1353386	A	0.94	(0.81, 1.09)	0.41	1
6	*CASC15*	rs10946507	A	0.97	(0.81, 1.16)	0.71	1
6	*CASC15*	rs4712652	G	1.06	(0.87, 1.28)	0.59	1
15	*FBN1*	rs25458	G	0.98	(0.86, 1.12)	0.75	1
15	*FBN1*	rs9806595	C	1.02	(0.9, 1.16)	0.76	1
2	*FMNL2*	rs1579050	G	1.61	(1.16, 2.25)	0.0049	0.08
6	*NHSL1*	rs4620141	C	0.95	(0.84, 1.08)	0.44	1
6	*NHSL1*	rs4896367	C	0.91	(0.8, 1.03)	0.13	1
4	*PDGFRA*	rs17084051	A	1.13	(0.97, 1.32)	0.11	1
4	*PDGFRA*	rs2114039	C	1.14	(1.00, 1.31)	0.05	0.8
4	*PDGFRA*	rs2228230	T	1.04	(0.87, 1.23)	0.70	1
1	*RPS6KC1*	rs12144639	A	1.03	(0.89, 1.19)	0.69	1
2	*TRAF3IP1*	rs77008212	G	1.26	(0.94, 1.69)	0.12	1
1	*ZC3H11B*	rs7525202	G	0.99	(0.86, 1.13)	0.84	1

SNP, single-nucleotide polymorphisms; Chr, chromosome; A1, effect allele; A2, reference allele; OR, odds ratio; CI, confidence interval* P value adjusted for participant age and sex^#^ Pc: Corrected P value based on P value times 16 (total number of tests)Footnote: all genetic associations are reported in additive model

**Table 2:** Allelic associations of SNPs with magnitude of Corneal Astigmatism in 2,167 children.

Chr	Gene/Locus	SNP	A1	β	95% CI	*P*	Pc^#^
4	*BMP3*	rs1353386	A	-0.003	(-0.054, 0.047)	0.89	1
6	*CASC15*	rs10946507	A	-0.019	(-0.079, 0.042)	0.55	1
6	*CASC15*	rs4712652	G	0.029	(-0.035, 0.094)	0.37	1
15	*FBN1*	rs25458	G	0.009	(-0.034, 0.052)	0.68	1
15	*FBN1*	rs9806595	C	0.014	(-0.029, 0.058)	0.51	1
2	*FMNL2*	rs1579050	G	0.158	(0.055, 0.262)	0.0028	**0.045**
6	*NHSL1*	rs4620141	C	-0.022	(-0.062, 0.019)	0.29	1
6	*NHSL1*	rs4896367	C	-0.015	(-0.058, 0.028)	0.49	1
4	*PDGFRA*	rs17084051	A	0.029	(-0.021, 0.079)	0.26	1
4	*PDGFRA*	rs2114039	C	0.026	(-0.018, 0.071)	0.24	1
4	*PDGFRA*	rs2228230	T	0.036	(-0.022, 0.094)	0.23	1
1	*RPS6KC1*	rs12144639	A	-0.019	(-0.068, 0.03)	0.45	1
2	*TRAF3IP1*	rs77008212	G	0.050	(-0.045, 0.145)	0.30	1
1	*ZC3H11B*	rs7525202	G	0.025	(-0.021, 0.07)	0.28	1

SNP, single-nucleotide polymorphisms; Chr, chromosome; A1, effect allele; A2, reference allele; CI, confidence interval* P value adjusted for participant age^#^ Pc: Corrected P value based on P value times 16 (total number of tests)Footnote: all genetic associations are reported in additive model

### Associations of SNPs with the risk of significant CA and magnitude of CA in boys and girls

Tables S3 and S4 show the allelic associations of SNPs with the risk of significant CA and magnitude of CA in boys and girls, respectively. The subgroup analyses revealed no significant sex-specific association between any SNP and the risk of significant CA or magnitude of CA in boys or girls.

### Associations of SNPs with the risk of significant CA and magnitude of CA across different age groups

Tables 3 and 4 show the allelic associations of SNPs with the risk of significant CA and magnitude of CA, respectively, across age quartiles. *PDGFRA* rs17084051 (*P* for linear trend = 0.04) and *ZC3H11B* rs7525202 (*P* for linear trend = 0.01) were significantly associated with the linear decrease in magnitude of CA across age quartiles.

**Table 3:** Allelic associations with the risk of significant Corneal Astigmatism (≥ 1.0D) across age quartiles.

Chr	Gene/ Locus	SNP	A1	Age quartile 1	Age quartile 2	Age quartile 3	Age quartile 4	*P*
				OR	OR	OR	OR	
4	*BMP3*	rs1353386	A	1.02	0.74	0.80	1.34	0.51
6	*CASC15*	rs10946507	A	0.96	0.98	1.04	0.91	0.84
6	*CASC15*	rs4712652	G	1.03	1.26	1.07	0.93	0.53
15	*FBN1*	rs25458	G	0.96	1.05	0.98	0.92	0.54
15	*FBN1*	rs9806595	C	1.11	1.01	1.03	0.94	0.84
2	*FMNL2*	rs1579050	G	1.21	1.12	1.78	2.66	0.09
6	*NHSL1*	rs4620141	C	0.81	1.28	0.88	0.91	0.94
6	*NHSL1*	rs4896367	C	1.02	0.81	1.02	0.81	0.57
4	*PDGFRA*	rs17084051	A	1.17	1.32	1.21	0.90	0.34
4	*PDGFRA*	rs2114039	C	1.25	1.29	1.31	0.83	0.29
4	*PDGFRA*	rs2228230	T	1.24	1.07	1.12	0.78	0.12
1	*RPS6KC1*	rs12144639	A	1.02	1.02	1.20	0.89	0.77
2	*TRAF3IP1*	rs77008212	G	0.69	2.00	1.63	1.33	0.64
1	*ZC3H11B*	rs7525202	G	1.05	1.07	0.96	0.90	0.08

SNP, single-nucleotide polymorphisms; Chr, chromosome; A1, effect allele; A2, reference allele; CI, confidence interval.

* P value adjusted for participant age and sex.

Footnote: all genetic associations are reported in additive model.

**Table 4:** Allelic associations with the magnitude of Corneal Astigmatism across age quartiles.

Chr	Gene/ Locus	SNP	A1	Q1 (*n* = 543)	Q2 (*n* = 543)	Q3 (*n* = 541)	Q4 (*n* = 540)	*P*
				β	β	β	β	
4	*BMP3*	rs1353386	A	0.219	-0.053	-0.062	0.045	0.48
6	*CASC15*	rs10946507	A	-0.174	0.113	-0.011	-0.051	0.73
6	*CASC15*	rs4712652	G	-0.143	0.082	0.069	0.023	0.40
15	*FBN1*	rs25458	G	0.228	0.048	0.041	-0.044	0.07
15	*FBN1*	rs9806595	C	0.282	0.036	0.015	-0.037	0.11
2	*FMNL2*	rs1579050	G	-0.045	0.214	0.101	0.301	0.20
6	*NHSL1*	rs4620141	C	-0.006	0.060	-0.045	-0.072	0.31
6	*NHSL1*	rs4896367	C	0.270	-0.040	-0.038	-0.005	0.29
4	*PDGFRA*	rs17084051	A	0.138	0.064	0.038	0.014	**0.04**
4	*PDGFRA*	rs2114039	C	0.088	0.056	0.067	-0.032	0.15
4	*PDGFRA*	rs2228230	T	-0.045	0.054	0.052	-0.014	0.76
1	*RPS6KC1*	rs12144639	A	-0.145	-0.054	0.057	-0.024	0.27
2	*TRAF3IP1*	rs77008212	G	-0.176	0.231	0.122	0.013	0.66
1	*ZC3H11B*	rs7525202	G	0.140	0.062	0.024	-0.021	**0.01**

SNP, single-nucleotide polymorphisms; Chr, chromosome; A1, effect allele; A2, reference allele; CI, confidence interval.

* P value adjusted for participant age and sex.

Footnote: all genetic associations are reported in additive model.

*FMNL2* rs1579050 was associated with the magnitude of CA (β = 0.301D, 95% CI = 0.116–0.485, *P* = 0.0015, *Pc* = 0.02) in age quartile 4 (Table 5). Tables S5, S6 and S7 show the allelic associations with risk of significant CA and magnitude of CA in age quartiles 1, 2 and 3, respectively. However, no significant associations were found. Tables S8 and S9 showed genotypic associations of *FMNL2* rs1579050 in dominant and recessive models. Under a dominant model, *FMNL2* rs1579050 was associated with magnitude of CA in age quartile 4 (β = 0.3D, 95% CI = 0.103–0.497, *P* = 0.0029, *Pc* = 0.04). There were also borderline associations with overall risk of significant CA (OR = 1.68, 95% CI = 1.19–2.38, *P* = 0.0033, *Pc* = 0.053), and in the age quartile 4 (OR = 3.08, 95% CI = 1.45–6.55, *P* = 0.0034, *Pc* = 0.054).

**Table 5:** Allelic associations with the risk of significant Corneal Astigmatism (≥ 1.0D) and magnitude of Corneal Astigmatism in age quartile 4.

Chr	Gene/ Locus	SNP	A1	Risk of significant corneal astigmatism				Magnitude of corneal astigmatism			
				OR	95% CI	*P*	Pc^#^	β	95% CI	*P*	Pc^#^
4	*BMP3*	rs1353386	A	1.34	(0.98, 1.83)	0.07	0.94	0.045	(-0.052, 0.142)	0.36	1
6	*CASC15*	rs10946507	A	0.91	(0.63, 1.33)	0.64	1	-0.051	(-0.171, 0.068)	0.40	1
6	*CASC15*	rs4712652	G	0.93	(0.62, 1.38)	0.71	1	0.023	(-0.104, 0.149)	0.73	1
15	*FBN1*	rs25458	G	0.92	(0.71, 1.19)	0.54	1	-0.044	(-0.125, 0.038)	0.29	1
15	*FBN1*	rs9806595	C	0.94	(0.72, 1.23)	0.67	1	-0.037	(-0.121, 0.047)	0.39	1
2	*FMNL2*	rs1579050	G	2.66	(1.31, 5.4)	0.01	0.16	0.301	(0.116, 0.485)	0.0015	**0.02**
6	*NHSL1*	rs4620141	C	0.91	(0.71, 1.17)	0.46	1	-0.072	(-0.151, 0.006)	0.07	1
6	*NHSL1*	rs4896367	C	0.81	(0.62, 1.06)	0.12	1	-0.005	(-0.091, 0.081)	0.91	1
4	*PDGFRA*	rs17084051	A	0.90	(0.67, 1.21)	0.50	1	0.014	(-0.08, 0.107)	0.78	1
4	*PDGFRA*	rs2114039	C	0.83	(0.64, 1.08)	0.16	1	-0.032	(-0.114, 0.051)	0.45	1
4	*PDGFRA*	rs2228230	T	0.78	(0.55, 1.09)	0.15	1	-0.014	(-0.123, 0.096)	0.81	1
1	*RPS6KC1*	rs12144639	A	0.89	(0.67, 1.17)	0.40	1	-0.024	(-0.113, 0.065)	0.59	1
2	*TRAF3IP1*	rs77008212	G	1.33	(0.79, 2.25)	0.29	1	0.013	(-0.15, 0.176)	0.88	1
1	*ZC3H11B*	rs7525202	G	0.90	(0.69, 1.18)	0.43	1	-0.021	(-0.107, 0.065)	0.64	1

SNP, single-nucleotide polymorphisms; Chr, chromosome; A1, effect allele; A2, reference allele; CI, confidence interval.

* P value adjusted for participant age and sex.

^#^ Pc: Corrected P value based on P value times 16 (total number of tests).

Footnote: All genetic associations are reported in additive model.

## Discussion

To the best of our knowledge, this is the first genetic association study of CA in children. In this study, we investigated the associations of 14 SNPs with the risk of significant CA and magnitude of CA in children aged 4–11 years. There were several notable findings. *FMNL2* rs1579050 was associated with CA magnitude. *PDGFRA* rs17084051 and *ZC3H11B* rs7525202 were associated with linear decrease in CA magnitude across age quartiles. The differences in the association of SNPs in relation to age highlights the potential age-specific effects on the genetic effects of CA. The associations reported in this study intend to contribute to the broader goal of developing polygenic risk scores or risk predictions based on individual’s genetic profiles.

*FMNL2* rs1579050 has been associated with CA in adults from previous GWAS. In our children cohort, the G allele of *FMNL2* rs1579050 was associated with increased CA magnitude. Interestingly, the alternative A allele has been associated with an increased magnitude of CA in adult Europeans in GWAS. The difference in the direction of effect could be explained by the ethnical difference, or an age-dependent effect of this SNP. The *FMNL2* gene encodes for formin-related proteins that are essential for cellular processes like cytokinesis and cell polarity^25^. The precise biological mechanisms linking *FMNL2* to CA remain unclear; however, its established role in cytoskeleton and its associations with other ocular phenotypes – including intraocular pressure^26^, primary open angle glaucoma^27^ and vertical cup-to-disc ratio^28^– suggests broader implications for ocular tissue morphology and remodeling rather than a glaucoma-specific mechanism alone. Further research should explore the pathways through which *FMNL2* influences the risk and magnitude of CA in the pediatric population.

In our cohort, the A allele of *PDGFRA* rs17084051 was associated with linear decrease in CA magnitude across age quartiles. This SNP has been associated with CA in Asian population^21^ and CC in multiple populations^19,22,29^. *PDGFRA* encodes for platelet derived growth factor receptor alpha, a cell surface tyrosine kinase receptor, that is expressed in human cornea^30^. It is well-known for its role in CC^19,21,22^, and has been associated with CA in GWAS^16^ and replicated in a Japanese population^23^. Taken together, the previous research and our findings suggested the role of *PDGFRA* may be in the coordinated growth and flattening of cornea during emmetropization, leading secondarily to astigmatism, highlighting its potential involvement of *PDGFRA* in the biological mechanisms underlying the development of CA.

Proxy SNP *ZC3H11B* rs7525202 was associated with refractive error in European populations^31^, while the original SNP *ZC3H11B* rs12032649 was associated CA in UK Europeans^15^. Rs7525202 A allele was correlated with rs12032649 G allele, while rs7525202 G allele was correlated with T allele. In our cohort, G allele of rs7525202 was associated with decreasing CA magnitude with age, which means the direction of effect of G allele went from risk to protective, aligning with that in adult GWAS. *ZC3H11B* is a gene that codes for zinc finger CCCH domain-containing protein 11B. It has regulatory functions including metal ion binding and poly(A)+ mRNA transport and is expressed in ocular tissues such as sclera and neural retina^32^. While its exact role and functions are still under investigation, it is plausible that this gene is involved in the regulation of mRNA of cornea-related genes by inhibiting their transcription, influencing CA phenotype.

Several limitations of the study exist. First, the effect sizes of SNPs identified are generally small. While this aligns with the polygenic nature of the genetic architecture of CA, a larger sample size may be required in replication to increase statistical power and for the robust validation of the significant associations. The results of low-frequency variants should be approached with caution, as a larger sample size may be required to include more participants carrying the effect allele, and to confirm its association with CA. Second, as our cohort consist of Hong Kong Chinese participants, the generalizability of the associations in this study to populations of a different age or ethnicity is unknown. Further replication studies should be conducted to validate these associations in Asian children and compare the effects of these SNPs in different age or ethnic populations. Third, since the study-wide and nominally associated SNPs are intronic variants, their roles and functions in CA should be interpreted with the knowledge that these SNPs are not on protein-coding sequences. These SNPs may play regulatory roles in the relevant biological processes instead, necessitating evidence from functional studies to explore the correlated pathways and molecules. There is a possibility of false positives within the detected associations. Fourth, environmental and behavioral factors that possibly contribute to CA formation was not taken in account, so the interaction between genetic and environmental factors in this multifactorial condition was not explored. Fifth, parental astigmatism was not accounted for in this study. As astigmatism can be highly heritable, future studies can adopt a family-based approach with parental phenotyping included to address this gap. Sixth, our analyses focused mainly on the risk and magnitude of CA and did not incorporate axis or full vector representations, which may have distinct genetic mechanisms from those influencing risk and magnitude and should be explored in future studies. Finally, corneal measurements were only obtained after cycloplegia, any related changes in corneal shape cannot be directly assessed of their effect.

In this study, we identified four possible genetic loci for increased risk and/or magnitude of corneal astigmatism in Chinese children. *FMNL2* is associated with the magnitude of CA; *PDGFRA* and *ZC3H11B* are associated with linear decrease in magnitude with age. We introduced possible novel associations of CA in children, which revealed shared genetic markers between CA and CC, between adults and children, and between different populations. Further genetic association studies involving a larger sample size in children are needed to validate these associations of SNPs with the risk and magnitude of CA. Nevertheless, our study demonstrated the variability of genetic association at different ages of a child and leads us to investigate further the role of these intronic variants in the development of astigmatism in childhood. These findings support the development of genetic models that assess the risk and magnitude of CA in children by integrating their genetic information and age. As more risk factors for child astigmatism are unveiled, such models may eventually complement routine refractive screening by identifying children at higher risk of developing severe astigmatism and prioritizing them for closer monitoring.

## Methods

### Study subjects and phenotype measurements

Children aged 4–11 years were recruited from the Hong Kong Children Eye Study (HKCES), a population-based and school-based study commenced in 2015, investigating the occurrence and development of pediatric ocular diseases. Comprehensive ophthalmic examinations were performed on all participants and information regarding lifestyle habits was collected via standardized questionnaires. The study protocol was previously published, with sample selection completed based on a stratified and clustered randomized sampling frame^33–36^. Children with congenital diseases, prior ocular trauma and surgery were excluded from this study. Ethics approval was obtained from the Ethics Committee of the Chinese University of Hong Kong. Written informed consent was obtained from participating children and their parents. All procedures were performed in accordance with the tenets of the Declaration of Helsinki^37^.

Ocular biometrics of participants were measured after a cycloplegic regimen of at least two cycles of eye drops. Each cycle of eye drops consists of 1% cyclopentolate and 1% tropicamide. The two cycles of eye drops were administered 10 min apart, with the addition of a third cycle if the child’s pupil size was ≤ 6.0 mm or the pupillary reflex was still present. Ocular biometric measurements were taken 30 min after the last cycle.

After cycloplegia, corneal curvatures at the flattest (K1) and steepest (K2) meridians were measured with a Zeiss IOL Master optical biometer (Carl Zeiss Meditec, Jena, Germany). Spherical power was measured using an ARK-510A autorefractor (Nidek, San Jose, CA). The magnitude of CA was calculated by the absolute difference between K1 and K2.

### Candidate SNP selection and genotyping

A total of 14 SNPs in 9 genes/loci were selected from genes or loci with overlapped associations with CA-related phenotypes in adult populations, reported in GWAS^24^: *BMP3* rs1353386, *CASC15* rs4712652 and rs10946507, *FBN1* rs35026266 and rs9806595, *FMNL2* rs1579050, *NHSL1* rs4620141 and rs4896367, *PDGFRA* rs17084051, rs2114039 and rs2228230, *RPS6KC1* rs12144639, *TRAF3IP1* rs77008212, and *ZC3H11B* rs12032649. Five SNPs are categorized as intergenic variants (*BMP3* rs1353386, *CASC15* rs4712652 and rs10946507, *RPS6KC1* rs12144639 and *ZC3H11B* rs7525202). Eight SNPs were intronic variants (*FBN1* rs35026266 and rs9806595, *FMNL2* rs1579050, *NHSL1* rs4620141 and rs4896367, *PDGFRA* rs17084051, rs2114039, and *TRAF3IP1* rs77008212). Despite being located outside the coding region, both intergenic and intronic variants can contribute to genetic diversity and disease susceptibility through RNA splicing and regulation of gene expression^38^. *PDGFRA* rs2228230 is the only synonymous variant that exists within the coding region but does not alter the sequence of the amino acid being produced, thus a minimal impact on the function of the gene^39^. None of these SNPs were identified in our previous reports evaluating the genetic associations with spherical value, spherical equivalent^40^, or anisometropia^41^.

Candidate SNPs were selected according two main rounds of selection, first according to the criteria that the locus or gene had overlapping associations between RA, CA and CC with a *P* value of less than 1 ⋅ 10^− 5^ from previous external GWAS reports^15,16,18–21^. Second, for SNPs in high linkage disequilibrium (LD; r^2^ ≥ 0.8), only one SNP was chosen to effectively capture the association of the entire LD block. The following criteria were applied to select the representative SNP: (1) the strongest associated SNP in the LD block, or (2) the SNP that is also associated with other ocular phenotypes e.g., myopia, spherical equivalent refraction (SER), axial length (AL). SNPs with effect allele frequency of < 0.01 were excluded. *FBN1* rs25458 was selected as a substitute as it is in complete LD (r^2^ = 1.0) with the original SNP rs35026266, and *ZC3H11B* rs7525202 was a substitute in complete LD with the original SNP rs12032649, since both original SNPs were not commercially available.

Genomic DNA samples were extracted from subjects’ peripheral blood or buccal swabs using the QIAamp DNA kit (Qiagen, Hilden, Germany). Quality check was performed using NanoDrop to ensure quantity (> 10ng/µL) and purity (A260/A280: 1.8-2.0, A260/A230: 2.0-2.2) for downstream analysis. All 14 candidate SNPs were genotyped using TaqMan SNP assays (Applied Biosystems, Foster City, CA) on a Light Cycler 480 Real-Time PCR System (Roche Diagnostics, Basel, Switzerland)^41^.

### Statistical analysis

Hardy-Weinberg equilibrium (HWE) test was performed for each SNP prior to the start of association analysis, where a SNP would be excluded from further analysis if its HWE *P* value was < 0.05. Magnitude of CA was defined by the absolute difference between K1 and K2. For binary CA, ‘significant CA’ is defined as CA ≥ 1.0D, as 1.0D is the common threshold requiring correction for clear vision. Sensitivity analyses using 0.75D and 1.5D threshold were also performed to confirm threshold-independent association (Tables S10 and S11). Data from right eye was used for analyses in this study to avoid selection bias for astigmatic eye (Pearson’s r for left and right eye CA values: 0.795). Associations of risk of significant CA with the candidate SNPs were estimated through multivariate logistic regression, with the results being reported as odds ratios (ORs) with corresponding 95% confidence intervals (CIs). The magnitude of CA was examined as a continuous variable and its associations with the candidate SNPs were evaluated with multivariable linear regression. Results were reported in β coefficients with 95% CI. Subgroup analysis was conducted for sex (female and male) and age, to assess variability of genetic associations across these demographic groups. Age was divided into quartiles. All regression analyses were adjusted for participant age and sex, with an exception that the stratifying variable (age and sex) was not included as a covariate in the corresponding subgroup analyses. Sensitivity analysis adjusting additionally for AL and spherical value was performed to address potential confounding by overall refractive status (Table S12). Bonferroni correction was applied for multiple testing, where the corrected *P* value (Pc) was given by the raw P value by 16, which is the total number of tests in the analysis, given by tests using additive models of 14 SNPs, and additional dominant and recessive models for the SNP *FMNL2* rs1579050 only due to low MAF (< 0.05). Pc < 0.05 is considered statistically significant. Additive genetic model only was reported for genetic associations with normal MAF to avoid overfitting of data and model selection bias in multiple-model genetic testing. A test for linear trend of allelic associations with the risk of significant CA or magnitude of CA across the age quartiles was performed by treating the age groups as ordinal variables. A *P* < 0.05 for the trend test was considered statistically significant.

## Supplementary Information

Below is the link to the electronic supplementary material.

Supplementary Material 1

## Data Availability

Deidentified participant data are available upon reasonable request from the corresponding authors.
