# Supplementary material for: Genetic associations of corneal astigmatism in Hong Kong Chinese children
Source: Sci Rep. 2026 Mar 31;16:15536. doi: 10.1038/s41598-026-46723-3 (PMC13187056; doi:10.1038/s41598-026-46723-3)
Supplement: Supplementary file 1 — Supplementary Material 1 [file 41598_2026_46723_MOESM1_ESM.pdf]

Supplementary Material for article named “Genetic Association of Corneal Astigmatism in Hong Kong Chinese Children: the Hong Kong Children Eye Study”

**Table S1. Demographics of study population**	**p. 1**
**Table S2. Genotyping results of SNPs in 2167 children**	**p. 2**
**Table S3. Allelic associations of SNPs with risk of significant CA in male and female**	**p. 3**
**Table S4. Allelic associations of SNPs with the magnitude of CA in male and female**	**p. 4**
**Table S5. Allelic associations with the risk of significant CA and magnitude of Corneal Astigmatism in age quartile 1**	**p. 5**
**Table S6. Allelic associations with the risk of significant CA and magnitude of Corneal Astigmatism in age quartile 2**	**p. 6**
**Table S7. Allelic associations with the risk of significant CA and magnitude of Corneal Astigmatism in age quartile 3**	**p. 7**
**Table S8. Dominant and recessive model for FMNL2 rs1579050 (effect allele: G) and risk of significant CA (including subgroups)**	**p. 8**
**Table S9. Dominant and recessive model for FMNL2 rs1579050 (effect allele: G) and magnitude of CA (including subgroups)**	**p. 9**
**Table S10. Sensitivity analysis of allelic associations with the risk of CA (≥0.75D)**	**p. 10**
**Table S11. Sensitivity analysis of allelic associations with the risk of CA (≥1.5D)**	**p. 11**
**Table S12. Sensitivity analysis with spherical value and axial length as covariates**	**p. 12**

Table S1. Demographics of study population

Characteristic	Overall (n=2167)	Control, CA < 1.0D (n=885)	Case, CA ≥ 1.0D (n=1282)	*P*
Age, years (mean ± SD)	7.67 ± 1.05	7.66 ± 1.03	7.67 ± 1.07	0.86
Sex, male (%)	1176 (54.3)	496 (56.0)	680 (53.0)	0.18
Corneal astigmatism (D)	1.21± 0.67	0.60 ± 0.24	1.57 ± 0.64	**< 0.001**
Height, cm	124.87 ± 8.19	124.97 ± 7.72	124.86 ± 8.47	0.75
Spherical power, D	0.54 ± 1.56	0.46 ± 1.23	0.59 ± 1.74	0.06
Axial length, mm	23.15 ± 0.93	23.25 ± 0.82	23.08 ± 1.00	**< 0.001**

Abbreviations: CA, corneal astigmatism; SD, standard deviation; D, diopters

Table S2. Genotyping results of SNPs in 2167 children

SNP	CHR	Location (GRCh37)	Gene/Locus	A1/A2	Genotype count	Call rate	HWE *P*	EAF
rs1353386	4	81947080	*BMP3*	A/C	85/690/1392	100%	1	0.20
rs10946507	6	22100367	*CASC15*	A/G	30/491/1646	100%	0.38	0.13
rs4712652	6	22078615	*CASC15*	G/A	19/452/1696	100%	0.07	0.11
rs25458	15	48797307	*FBN1*	G/A	218/949/1000	100%	0.77	0.32
rs9806595	15	48755168	*FBN1*	C/T	216/940/1011	100%	0.96	0.32
rs1579050	2	153364527	*FMNL2*	G/A	5/153/2009	100%	0.23	0.04
rs4620141	6	138869568	*NHSL1*	C/T	407/1083/677	100%	0.49	0.44
rs4896367	6	138807281	*NHSL1*	C/T	217/910/1040	100%	0.39	0.31
rs17084051	4	55087581	*PDGFRA*	A/C	89/679/1399	100%	0.54	0.20
rs2114039	4	55092626	*PDGFRA*	C/T	179/852/1136	100%	0.29	0.28
rs2228230	4	55152040	*PDGFRA*	T/C	41/511/1615	100%	0.93	0.14
rs12144639	1	213817311	*RPS6KC1*	A/G	101/725/1341	100%	0.80	0.21
rs77008212	2	239307113	*TRAF3IP1*	G/A	1/210/1956	100%	0.06	0.05
rs7525202	1	219788519	*ZC3H11B*	G/A	157/860/1150	100%	0.87	0.27

Abbreviations: SNP, single-nucleotide polymorphisms; CHR, chromosome; A1, effect allele; A2, reference allele; HWE, Hardy-Weinberg equilibrium; EAF, effect allele frequency

Table S3. Allelic associations of SNPs with risk of significant CA in male and female

Chr	Gene/ Locus	SNP	A1	Male (n=1176)				Female (n=991)			
				OR	95% CI	P	Pc	OR	95% CI	P	Pc
4	*BMP3*	rs1353386	A	0.92	(0.75, 1.14)	0.45	1	0.96	(0.77, 1.19)	0.68	1
6	*CASC15*	rs10946507	A	0.87	(0.68, 1.11)	0.25	1	1.10	(0.84, 1.45)	0.50	1
6	*CASC15*	rs4712652	G	0.97	(0.74, 1.26)	0.80	1	1.17	(0.88, 1.56)	0.28	1
15	*FBN1*	rs25458	G	1.02	(0.85, 1.21)	0.86	1	0.94	(0.77, 1.14)	0.52	1
15	*FBN1*	rs9806595	C	1.06	(0.88, 1.26)	0.55	1	0.98	(0.81, 1.19)	0.86	1
2	*FMNL2*	rs1579050	G	1.44	(0.91, 2.26)	0.12	1	1.85	(1.12, 3.06)	0.02	0.32
6	*NHSL1*	rs4620141	C	0.92	(0.78, 1.09)	0.35	1	0.99	(0.82, 1.19)	0.91	1
6	*NHSL1*	rs4896367	C	0.91	(0.76, 1.08)	0.27	1	0.91	(0.75, 1.1)	0.32	1
4	*PDGFRA*	rs17084051	A	1.05	(0.85, 1.29)	0.64	1	1.23	(0.98, 1.54)	0.07	1
4	*PDGFRA*	rs2114039	C	1.07	(0.89, 1.29)	0.45	1	1.23	(1.01, 1.5)	0.04	0.64
4	*PDGFRA*	rs2228230	T	0.91	(0.72, 1.14)	0.41	1	1.25	(0.95, 1.65)	0.12	1
1	*RPS6KC1*	rs12144639	A	0.98	(0.81, 1.2)	0.87	1	1.08	(0.87, 1.35)	0.47	1
2	*TRAF3IP1*	rs77008212	G	1.30	(0.87, 1.95)	0.20	1	1.22	(0.8, 1.86)	0.35	1
1	*ZC3H11B*	rs7525202	G	1.00	(0.83, 1.2)	0.99	1	0.97	(0.8, 1.19)	0.79	1

Abbreviations: SNP, single-nucleotide polymorphisms; Chr, chromosome; A1, effect allele; A2, reference allele; OR, odds ratio; CI, confidence interval

* P value adjusted for participant age and sex

[#] Pc: Corrected P value based on P value times 16 (total number of tests)

Footnote: All genetic associations are reported in additive model.

Table S4. Allelic associations of SNPs with the magnitude of CA in male and female

Chr	Gene/ Locus	SNP	A1	Male (n=1176)				Female (n=991)			
				β	95% CI	P	Pc#	β	95% CI	P	Pc#
4	*BMP3*	rs1353386	A	0.090	(-0.059, 0.238)	0.24	1	-0.020	(-0.09, 0.05)	0.58	1
6	*CASC15*	rs10946507	A	-0.092	(-0.266, 0.083)	0.30	1	0.028	(-0.06, 0.115)	0.54	1
6	*CASC15*	rs4712652	G	-0.038	(-0.228, 0.151)	0.69	1	0.050	(-0.041, 0.14)	0.28	1
15	*FBN1*	rs25458	G	0.136	(0.011, 0.26)	0.03	0.53	-0.007	(-0.069, 0.055)	0.82	1
15	*FBN1*	rs9806595	C	0.141	(0.015, 0.267)	0.03	0.45	0.005	(-0.056, 0.066)	0.87	1
2	*FMNL2*	rs1579050	G	0.172	(-0.138, 0.481)	0.28	1	0.108	(-0.035, 0.252)	0.14	1
6	*NHSL1*	rs4620141	C	-0.026	(-0.143, 0.09)	0.66	1	-0.003	(-0.062, 0.056)	0.92	1
6	*NHSL1*	rs4896367	C	0.093	(-0.031, 0.216)	0.14	1	-0.014	(-0.076, 0.047)	0.65	1
4	*PDGFRA*	rs17084051	A	0.082	(-0.064, 0.228)	0.27	1	0.051	(-0.019, 0.121)	0.16	1
4	*PDGFRA*	rs2114039	C	0.047	(-0.082, 0.176)	0.48	1	0.046	(-0.017, 0.108)	0.15	1
4	*PDGFRA*	rs2228230	T	-0.001	(-0.163, 0.162)	0.99	1	0.031	(-0.056, 0.118)	0.48	1
1	*RPS6KC1*	rs12144639	A	-0.066	(-0.207, 0.075)	0.36	1	-0.011	(-0.08, 0.059)	0.76	1
2	*TRAF3IP1*	rs77008212	G	0.015	(-0.266, 0.297)	0.92	1	0.048	(-0.084, 0.18)	0.47	1
1	*ZC3H11B*	rs7525202	G	0.053	(-0.078, 0.184)	0.43	1	0.043	(-0.021, 0.107)	0.19	1

Abbreviations: SNP, single-nucleotide polymorphisms; Chr, chromosome; A1, effect allele; A2, reference allele; CI, confidence interval

* P value adjusted for participant age

Pc: Corrected P value based on P value times 16 (total number of tests)

Footnote: all genetic associations are reported in additive model

Table S5. Allelic associations with the risk of significant CA and magnitude of Corneal Astigmatism in age quartile 1

Chr	Gene/ Locus	SNP	A1	Risk of corneal astigmatism				Magnitude of corneal astigmatism			
				OR	95% CI	P	Pc#	β	95% CI	P	Pc#
4	*BMP3*	rs1353386	A	1.02	(0.75, 1.38)	0.91	1	0.219	(-0.074, 0.511)	0.14	1
6	*CASC15*	rs10946507	A	0.96	(0.67, 1.36)	0.81	1	-0.174	(-0.516, 0.167)	0.32	1
6	*CASC15*	rs4712652	G	1.03	(0.71, 1.51)	0.87	1	-0.143	(-0.505, 0.22)	0.44	1
15	*FBN1*	rs25458	G	0.96	(0.74, 1.24)	0.77	1	0.228	(-0.019, 0.475)	0.07	1
15	*FBN1*	rs9806595	C	1.11	(0.85, 1.43)	0.45	1	0.282	(0.035, 0.529)	0.03	0.41
2	*FMNL2*	rs1579050	G	1.21	(0.63, 2.33)	0.57	1	-0.045	(-0.655, 0.566)	0.89	1
6	*NHSL1*	rs4620141	C	0.81	(0.63, 1.03)	0.08	1	-0.006	(-0.237, 0.226)	0.96	1
6	*NHSL1*	rs4896367	C	1.02	(0.78, 1.32)	0.91	1	0.270	(0.017, 0.524)	0.04	0.59
4	*PDGFRA*	rs17084051	A	1.17	(0.87, 1.57)	0.31	1	0.138	(-0.144, 0.42)	0.34	1
4	*PDGFRA*	rs2114039	C	1.25	(0.97, 1.62)	0.09	1	0.088	(-0.156, 0.333)	0.48	1
4	*PDGFRA*	rs2228230	T	1.24	(0.89, 1.75)	0.21	1	-0.045	(-0.367, 0.277)	0.78	1
1	*RPS6KC1*	rs12144639	A	1.02	(0.76, 1.38)	0.88	1	-0.145	(-0.432, 0.142)	0.32	1
2	*TRAF3IP1*	rs77008212	G	0.69	(0.39, 1.2)	0.18	1	-0.176	(-0.716, 0.363)	0.52	1
1	*ZC3H11B*	rs7525202	G	1.05	(0.8, 1.38)	0.73	1	0.140	(-0.124, 0.404)	0.30	1

Abbreviations: SNP, single-nucleotide polymorphisms; Chr, chromosome; A1, effect allele; A2, reference allele; CI, confidence interval

* P value adjusted for participant sex

Pc: Corrected P value based on P value times 16 (total number of tests)

Footnote: All genetic associations are reported in additive model.

Table S6. Allelic associations with the risk of significant CA and magnitude of Corneal Astigmatism in age quartile 2

Chr	Gene/ Locus	SNP	A1	Risk of corneal astigmatism				Magnitude of corneal astigmatism			
				OR	95% CI	P	Pc#	β	95% CI	P	Pc#
4	*BMP3*	rs1353386	A	0.74	(0.54, 1)	0.05	0.78	-0.053	(-0.161, 0.055)	0.34	1
6	*CASC15*	rs10946507	A	0.98	(0.66, 1.44)	0.90	1	0.113	(-0.026, 0.251)	0.11	1
6	*CASC15*	rs4712652	G	1.26	(0.83, 1.93)	0.28	1	0.082	(-0.064, 0.228)	0.27	1
15	*FBN1*	rs25458	G	1.05	(0.81, 1.36)	0.73	1	0.048	(-0.044, 0.14)	0.31	1
15	*FBN1*	rs9806595	C	1.01	(0.78, 1.31)	0.93	1	0.036	(-0.055, 0.126)	0.44	1
2	*FMNL2*	rs1579050	G	1.12	(0.56, 2.25)	0.75	1	0.214	(-0.029, 0.457)	0.08	1
6	*NHSL1*	rs4620141	C	1.28	(1, 1.65)	0.05	0.85	0.060	(-0.028, 0.148)	0.18	1
6	*NHSL1*	rs4896367	C	0.81	(0.63, 1.03)	0.09	1	-0.040	(-0.128, 0.048)	0.38	1
4	*PDGFRA*	rs17084051	A	1.32	(0.95, 1.82)	0.09	1	0.064	(-0.048, 0.175)	0.26	1
4	*PDGFRA*	rs2114039	C	1.29	(0.97, 1.72)	0.08	1	0.056	(-0.043, 0.155)	0.27	1
4	*PDGFRA*	rs2228230	T	1.07	(0.73, 1.57)	0.73	1	0.054	(-0.079, 0.188)	0.43	1
1	*RPS6KC1*	rs12144639	A	1.02	(0.77, 1.36)	0.88	1	-0.054	(-0.155, 0.048)	0.30	1
2	*TRAF3IP1*	rs77008212	G	2.00	(0.98, 4.08)	0.06	0.91	0.231	(0.003, 0.459)	0.05	0.75
1	*ZC3H11B*	rs7525202	G	1.07	(0.81, 1.41)	0.64	1	0.062	(-0.036, 0.16)	0.21	1

Abbreviations: SNP, single-nucleotide polymorphisms; Chr, chromosome; A1, effect allele; A2, reference allele; CI, confidence interval

* P value adjusted for participant sex

Pc: Corrected P value based on P value times 16 (total number of tests)

Footnote: All genetic associations are reported in additive model.

Table S7. Allelic associations with the risk of significant CA and magnitude of Corneal Astigmatism in age quartile 3

Chr	Gene/ Locus	SNP	A1	Risk of corneal astigmatism				Magnitude of corneal astigmatism			
				**OR**	**95% CI**	**P**	**Pc#**	**β**	**95% CI**	**P**	**Pc#**
4	*BMP3*	rs1353386	A	0.80	(0.6, 1.08)	0.15	1	-0.062	(-0.16, 0.036)	0.22	1
6	*CASC15*	rs10946507	A	1.04	(0.73, 1.47)	0.83	1	-0.011	(-0.126, 0.104)	0.85	1
6	*CASC15*	rs4712652	G	1.07	(0.73, 1.55)	0.73	1	0.069	(-0.054, 0.192)	0.27	1
15	*FBN1*	rs25458	G	0.98	(0.75, 1.28)	0.89	1	0.041	(-0.048, 0.129)	0.37	1
15	*FBN1*	rs9806595	C	1.03	(0.79, 1.33)	0.85	1	0.015	(-0.072, 0.101)	0.74	1
2	*FMNL2*	rs1579050	G	1.78	(0.94, 3.4)	0.08	1	0.101	(-0.101, 0.304)	0.33	1
6	*NHSL1*	rs4620141	C	0.88	(0.69, 1.12)	0.30	1	-0.045	(-0.126, 0.035)	0.27	1
6	*NHSL1*	rs4896367	C	1.02	(0.79, 1.32)	0.86	1	-0.038	(-0.122, 0.046)	0.37	1
4	*PDGFRA*	rs17084051	A	1.21	(0.89, 1.64)	0.22	1	0.038	(-0.062, 0.139)	0.46	1
4	*PDGFRA*	rs2114039	C	1.31	(0.99, 1.72)	0.06	0.94	0.067	(-0.023, 0.157)	0.14	1
4	*PDGFRA*	rs2228230	T	1.12	(0.78, 1.6)	0.53	1	0.052	(-0.065, 0.169)	0.38	1
1	*RPS6KC 1*	rs12144639	A	1.20	(0.87, 1.66)	0.26	1	0.057	(-0.048, 0.162)	0.28	1
2	*TRAF3IP 1*	rs77008212	G	1.63	(0.88, 3.03)	0.12	1	0.122	(-0.075, 0.319)	0.23	1
1	*ZC3H11B*	rs7525202	G	0.96	(0.74, 1.26)	0.78	1	0.024	(-0.065, 0.113)	0.60	1

Abbreviations: SNP, single-nucleotide polymorphisms; Chr, chromosome; A1, effect allele; A2, reference allele; CI, confidence interval

* P value adjusted for participant age and sex

Pc: Corrected P value based on P value times 16 (total number of tests)

Footnote: All genetic associations are reported in additive model.

Table S8. Dominant and recessive model for *FMNL2* rs1579050 (effect allele: G) and risk of significant CA (including subgroups)

Risk of CA (≥ 1.0D)	Model	OR	95% CI	P *	Pc #
All participants	Dominant	1.68	(1.19, 2.38)	0.0033	0.053
	Recessive	1.07	(0.18, 6.42)	0.94	1
Boys only	Dominant	1.42	(0.9, 2.24)	0.13	1
	Recessive	NA	NA	NA	NA
Girls only	Dominant	2.10	(1.23, 3.61)	0.007	0.11
	Recessive	0.68	(0.09, 4.82)	0.70	1
Age quartile 1	Dominant	1.25	(0.6, 2.6)	0.55	1
	Recessive	1.24	(0.11, 13.81)	0.86	1
Age quartile 2	Dominant	1.12	(0.56, 2.25)	0.75	1
	Recessive	NA	NA	NA	NA
Age quartile 3	Dominant	1.78	(0.94, 3.4)	0.08	1
	Recessive	NA	NA	NA	NA
Age quartile 4	Dominant	3.08	(1.45, 6.55)	0.0034	0.054
	Recessive	0.73	(0.05, 11.77)	0.83	1

Abbreviations: CA, corneal astigmatism; OR, odds ratio; CI, confidence interval
* P value adjusted for participant age and sex
# Pc: Corrected P value based on P value times 16 (total number of tests)

Table S9. Dominant and recessive model for *FMNL2* rs1579050 (effect allele: G) and magnitude of CA (including subgroups)

Magnitude of CA	Model	β	95% CI	P *	Pc #
All participants	Dominant	0.163	(0.054, 0.272)	0.0034	0.054
	Recessive	0.324	(-0.267, 0.915)	0.28	1
Boys only	Dominant	0.153	(-0.162, 0.468)	0.34	1
	Recessive	1.978	(-0.82, 4.776)	0.17	1
Girls only	Dominant	0.131	(-0.024, 0.286)	0.10	1
	Recessive	-0.068	(-0.708, 0.572)	0.84	1
Age quartile 1	Dominant	-0.055	(-0.745, 0.635)	0.88	1
	Recessive	-0.016	(-2.245, 2.213)	0.99	1
Age quartile 2	Dominant	0.214	(-0.029, 0.457)	0.08	1
	Recessive	NA	NA	NA	NA
Age quartile 3	Dominant	0.101	(-0.101, 0.304)	0.33	1
	Recessive	NA	NA	NA	NA
Age quartile 4	Dominant	0.300	(0.103, 0.497)	0.0029	**0.04**
	Recessive	0.862	(-0.029, 1.752)	0.06	0.82

Abbreviations: CA, corneal astigmatism; CI, confidence interval
* P value adjusted for participant age and sex
# Pc: Corrected P value based on P value times 16 (total number of tests)

Table S10. Sensitivity analysis of allelic associations with the risk of CA (≥0.75D)

Chr	Gene/Locus	SNP	A1	Model	OR	95% CI	P	Pc[#]
4	*BMP3*	rs1353386	A	Additive	1.08	(0.91, 1.29)	0.38	1
6	*CASC15*	rs10946507	A	Additive	1.09	(0.88, 1.35)	0.43	1
6	*CASC15*	rs4712652	G	Additive	1.09	(0.87, 1.37)	0.46	1
15	*FBN1*	rs25458	G	Additive	1.01	(0.87, 1.17)	0.93	1
15	*FBN1*	rs9806595	C	Additive	1.02	(0.88, 1.18)	0.82	1
2	*FMNL2*	rs1579050	G	Additive	1.92	(1.23, 3)	0.004	0.064
				Dominant	1.91	(1.21, 3.01)	0.005	0.16
				Recessive	NA	NA	NA	NA
6	*NHSL1*	rs4620141	C	Additive	0.98	(0.85, 1.13)	0.79	1
6	*NHSL1*	rs4896367	C	Additive	0.87	(0.75, 1.01)	0.07	1
4	*PDGFRA*	rs17084051	A	Additive	1.08	(0.91, 1.29)	0.38	1
4	*PDGFRA*	rs2114039	C	Additive	1.11	(0.95, 1.3)	0.19	1
4	*PDGFRA*	rs2228230	T	Additive	1.05	(0.86, 1.29)	0.64	1
1	*RPS6KC1*	rs12144639	A	Additive	1.08	(0.91, 1.28)	0.39	1
2	*TRAF3IP1*	rs77008212	G	Additive	1.17	(0.83, 1.66)	0.36	1
1	*ZC3H11B*	rs7525202	G	Additive	0.94	(0.81, 1.1)	0.47	1

Abbreviations: SNP, single-nucleotide polymorphisms; Chr, chromosome; A1, effect allele; A2, reference allele; CI, confidence interval

* P value adjusted for participant age

[#] Pc: Corrected P value based on P value times 16 (total number of tests)

Table S11. Sensitivity analysis of allelic associations with the risk of CA (≥ 1.5 D)

Chr	Gene/Locus	SNP	A1	Model	OR	95% CI	P	Pc[#]
4	*BMP3*	rs1353386	A	Additive	0.95	(0.8, 1.13)	0.56	1
6	*CASC15*	rs10946507	A	Additive	0.99	(0.8, 1.22)	0.91	1
6	*CASC15*	rs4712652	G	Additive	1.03	(0.82, 1.28)	0.81	1
15	*FBN1*	rs25458	G	Additive	0.96	(0.82, 1.11)	0.56	1
15	*FBN1*	rs9806595	C	Additive	0.99	(0.86, 1.15)	0.92	1
2	*FMNL2*	rs1579050	G	Additive	1.56	(1.12, 2.16)	0.008	0.16
				Dominant	1.64	(1.16, 2.32)	0.005	0.08
				Recessive	0.74	(0.08, 6.67)	0.791	1
6	*NHSL1*	rs4620141	C	Additive	0.95	(0.83, 1.1)	0.52	1
6	*NHSL1*	rs4896367	C	Additive	0.95	(0.82, 1.1)	0.51	1
4	*PDGFRA*	rs17084051	A	Additive	1.08	(0.91, 1.28)	0.39	1
4	*PDGFRA*	rs2114039	C	Additive	1.08	(0.93, 1.25)	0.32	1
4	*PDGFRA*	rs2228230	T	Additive	1.18	(0.97, 1.44)	0.09	1
1	*RPS6KC1*	rs12144639	A	Additive	0.95	(0.8, 1.13)	0.58	1
2	*TRAF3IP1*	rs77008212	G	Additive	1.25	(0.92, 1.71)	0.16	1
1	*ZC3H11B*	rs7525202	G	Additive	1.07	(0.92, 1.25)	0.38	1

Abbreviations: SNP, single-nucleotide polymorphisms; Chr, chromosome; A1, effect allele; A2, reference allele; CI, confidence interval

* P value adjusted for participant age

[#] Pc: Corrected P value based on P value times 16 (total number of tests)

Table S12. Sensitivity analysis with spherical value and axial length as covariates

Chr	Gene/Locus	SNP	A1	Model	OR	95% CI	P	Pc#
4	*BMP3*	rs1353386	A	Additive	0.95	(0.81, 1.1)	0.48	1
6	*CASC15*	rs10946507	A	Additive	0.96	(0.8, 1.16)	0.7	1
6	*CASC15*	rs4712652	G	Additive	1.06	(0.87, 1.3)	0.53	1
15	*FBN1*	rs25458	G	Additive	0.99	(0.87, 1.13)	0.89	1
15	*FBN1*	rs9806595	C	Additive	1.04	(0.91, 1.19)	0.57	1
2	*FMNL2*	rs1579050	G	Additive	1.64	(1.17, 2.29)	0.004	0.064
				Dominant	1.71	(1.2, 2.43)	0.0029	**0.046**
				Recessive	1.19	(0.2, 7.18)	0.8482	1
6	*NHSL1*	rs4620141	C	Additive	0.96	(0.85, 1.09)	0.51	1
6	*NHSL1*	rs4896367	C	Additive	0.91	(0.8, 1.03)	0.14	1
4	*PDGFRA*	rs17084051	A	Additive	1.11	(0.95, 1.3)	0.17	1
4	*PDGFRA*	rs2114039	C	Additive	1.13	(0.98, 1.29)	0.08	1
4	*PDGFRA*	rs2228230	T	Additive	1.02	(0.86, 1.22)	0.8	1
1	*RPS6KC1*	rs12144639	A	Additive	1.03	(0.89, 1.19)	0.72	1
2	*TRAF3IP1*	rs77008212	G	Additive	1.22	(0.91, 1.64)	0.18	1
1	*ZC3H11B*	rs7525202	G	Additive	0.97	(0.85, 1.11)	0.66	1

Abbreviations: SNP, single-nucleotide polymorphisms; Chr, chromosome; A1, effect allele; A2, reference allele; CI, confidence interval

* P value adjusted for participant age, sex, spherical value and axial length

Pc: Corrected P value based on P value times 16 (total number of tests)
